# Microbiological Spectrum and Antimicrobial Susceptibility Patterns of Culture‐Positive Thoracic and Respiratory Specimens in Syria: A Retrospective Laboratory‐Based Study

**DOI:** 10.1155/ijm/5705248

**Published:** 2026-10-02

**Authors:** Mohammad Alaa Aldakak, Alaa Senjab, Hussein Aljbawi, Ibrahim Alkhatib, Adnan Alkhatib, Bassam Darwish

**Affiliations:** ^1^ Faculty of Medicine Damascus University Damascus Syria damascusuniversity.edu.sy; ^2^ Faculty of Medicine Syrian Private University Damascus Syria spu.edu.sy; ^3^ Alkhatib Medical Laboratory Damascus Syria

**Keywords:** antimicrobial resistance, antimicrobial susceptibility, empyema thoracis, Syria, thoracic surgical infection

## Abstract

**Background:**

Thoracic and respiratory infections require timely microbiological diagnosis, whereas pathogen distribution and antimicrobial susceptibility may vary substantially across settings and specimen types. Local laboratory‐based data can therefore support antimicrobial‐resistance surveillance and interpretation of culture results.

**Methods:**

We conducted a retrospective laboratory‐based study of thoracic and respiratory culture records processed at a private referral microbiology laboratory in Damascus, Syria, from January 1, 2022, to December 31, 2025. Culture‐positive specimens were categorized as pleural‐space, deep pulmonary/lung surgical, or tracheal/airway specimens. Pleural‐space and deep pulmonary/lung surgical specimens were combined as the primary analysis group, whereas airway specimens were analyzed separately as an exploratory subgroup. Antimicrobial susceptibility testing was interpreted according to CLSI M100, 30th edition (2020), with agent‐specific tested denominators and cautious interpretation of estimates based on fewer than 30 isolates.

**Results:**

Among 356 eligible culture records, 136 were positive (38.2%). The mean age was 42.3 ± 21.5 years, and 66.9% were from male patients. Bacterial isolates accounted for 127/136 (93.4%). The most frequent bacteria were *Staphylococcus aureus* (38/127, 29.9%), *Escherichia coli* (30/127, 23.6%), *Klebsiella pneumoniae* (26/127, 20.5%), and *Pseudomonas aeruginosa* (18/127, 14.2%). In the primary pleural/deep group, *S*. *aureus* showed high in vitro susceptibility to vancomycin (35/35, 100%) and linezolid (33/35, 94.3%). Among dominant gram‐negative isolates, amikacin and meropenem showed comparatively high activity, whereas several cephalosporins, fluoroquinolones, and trimethoprim‐sulfamethoxazole showed lower susceptibility. No colistin‐resistant isolate was identified.

**Conclusion:**

Culture‐positive thoracic and respiratory specimens in this Syrian referral laboratory showed a mixed *S*. *aureus* and gram‐negative microbiological profile. These findings provide local laboratory‐based susceptibility signals that support organism‐specific AST interpretation and antimicrobial‐resistance surveillance, whereas small organism‐specific denominators preclude their use as formal empirical‐treatment recommendations.

Central Message

Culture‐positive thoracic and respiratory specimens showed a mixed *Staphylococcus aureus* and gram‐negative profile, with variable in vitro susceptibility, supporting organism‐specific antimicrobial susceptibility testing (AST) interpretation and local antimicrobial‐resistance surveillance.

## 1. Introduction

Thoracic and lower respiratory infections include several clinically important syndromes, particularly pleural‐space infection and empyema thoracis, as well as hospital‐acquired pneumonia (HAP) and ventilator‐associated pneumonia (VAP) [1, 2]. Empyema thoracis is a serious infection of the pleural space that may complicate pneumonia, pre‐existing pleural effusion, thoracic trauma, thoracic intervention, or thoracic surgery and is associated with substantial morbidity and mortality, particularly among elderly and chronically debilitated patients [2]. HAP and VAP also remain major acute‐care infections; HAP accounts for an important proportion of nosocomial infections, whereas VAP affects a considerable proportion of mechanically ventilated patients [1]. These infections require timely microbiological diagnosis because appropriate antimicrobial selection depends on identifying the responsible organisms and understanding their local susceptibility patterns [1, 2].

The microbiological spectrum of pleural and lower respiratory infections varies according to acquisition setting, geography, host characteristics, and prior antimicrobial exposure [2, 3]. Community‐acquired pleural infection is frequently dominated by streptococci and may also involve anaerobes, whereas hospital‐acquired or complicated pleural infection may show a greater contribution from gram‐negative organisms [2, 3]. Contemporary regional and hospital‐based studies have also demonstrated important roles for *Klebsiella pneumoniae*, *Escherichia coli*, *Pseudomonas aeruginosa*, and *Acinetobacter baumannii* in empyema and ICU‐associated respiratory infections [1, 2, 4]. Similarly, although lung abscess has traditionally been associated predominantly with anaerobic organisms, bacteriologically confirmed studies have demonstrated an important contribution from aerobic gram‐negative pathogens, particularly *K*. *pneumoniae* [5].

Fungal recovery adds further complexity to the interpretation of thoracic and respiratory cultures [6, 7]. *Candida* species may be recovered from respiratory specimens and are also recognized as normal‐flora organisms capable of opportunistic infection, making their clinical significance dependent on the host and specimen context [6, 8]. In contrast, *Aspergillus* species, particularly *Aspergillus fumigatus*, are established causes of invasive pulmonary disease in susceptible or critically ill patients and have been recovered from bronchoalveolar lavage and tracheal aspirate specimens in patients with probable pulmonary aspergillosis [7]. Accordingly, fungal isolates from thoracic or respiratory specimens should be interpreted in conjunction with specimen type, host factors, and available clinical and radiological findings [6–8].

Antimicrobial resistance further increases the importance of locally generated microbiological data. Antibiotic misuse, nonprescription access, incomplete treatment courses, and stewardship limitations have been reported in settings such as Nigeria and Syria and may contribute to antimicrobial selection pressure [2, 9]. Recent Syrian data have described widespread nonprescription antibiotic access, reliance on pharmacist‐directed antibiotic use, premature discontinuation after symptom improvement, and substantial use of Watch‐group antibiotics [9]. These observations reinforce the value of local susceptibility surveillance rather than relying solely on antimicrobial‐resistance patterns derived from other settings [2, 9]. Therefore, this study aimed to characterize the microbiological spectrum and organism‐specific in vitro antimicrobial susceptibility patterns of culture‐positive thoracic and respiratory specimens processed at a Syrian referral microbiology laboratory between 2022 and 2025. Primary susceptibility analyses focused on pleural‐space and deep pulmonary/lung surgical specimens, whereas tracheal/airway isolates were evaluated separately as an exploratory subgroup because the available laboratory data could not reliably distinguish infection from colonization.

## 2. Methods

### 2.1. Study Design, Setting, and Data Source

We conducted a retrospective laboratory‐based study of thoracic and respiratory culture records processed at Alkhatib, a private referral microbiology laboratory in Damascus, Syria, between January 1, 2022, and December 31, 2025. The laboratory receives referred specimens from multiple physicians, hospitals, and surgical services rather than from a single hospital catchment area. Specimens were submitted during routine clinical care for microbiological investigation of suspected thoracic or respiratory infection.

A total of 356 eligible culture records were identified during the study period and constituted the denominator for culture positivity. Of these, 136 were culture‐positive and formed the culture‐positive analytic cohort used for characterization of the microbiological spectrum and antimicrobial susceptibility patterns. Culture and AST data were extracted retrospectively from the laboratory information system in February 2026. Extracted variables included patient identifier, age, sex, specimen category, culture year, recovered microorganism, and available antimicrobial susceptibility results.

Because the database was laboratory‐based, detailed clinical variables, radiological findings, prior antimicrobial exposure, antimicrobial treatment regimens, operative findings, source‐control procedures, and clinical outcomes were not available. No missing data were present for age, sex, specimen category, culture year, or microorganism identification within the 136 culture‐positive records.

The study protocol was approved by the Ethics Committee of the Syrian Private University (Approval No. MD‐30032026‐140). The study was conducted in accordance with the principles of the Declaration of Helsinki and was reported in accordance with the Strengthening the Reporting of Observational Studies in Epidemiology (STROBE) statement for cross‐sectional studies.

### 2.2. Specimens and Eligibility Criteria

Eligible records comprised thoracic surgical and respiratory culture specimens processed during the study period. Culture‐positive specimens were categorized anatomically into three groups: pleural‐space specimens, deep pulmonary/lung surgical specimens, and tracheal/airway specimens.

Pleural‐space specimens included pleural fluid, pleural effusion, empyema samples, purulent pleural fluid, pleural secretions, pleural cavity secretions, pleural scraping, and pleural clots. The American Association for Thoracic Surgery consensus guidelines state that a positive pleural fluid culture establishes empyema in the appropriate clinical context [10]. Deep pulmonary/lung surgical specimens included pulmonary abscess samples or secretions, pulmonary tissue, pulmonary biopsy, and surgically obtained pulmonary secretions. Tracheal/airway specimens included tracheal secretions, tracheal tube specimens, and tracheal tube tips.

Patient identifiers were reviewed during data cleaning. Each culture‐positive record represented a unique patient, and no patient contributed more than one positive specimen during the study period. No polymicrobial cultures were identified in the final culture‐positive dataset; consequently, each positive specimen contributed one recovered microorganism. No patient contributed both a bacterial and a fungal isolate.

Pleural‐space and deep pulmonary/lung surgical specimens were combined as the primary pleural/deep surgical analysis group because these specimens originated from pleural or internal/deep sites. Tracheal/airway specimens were analyzed separately as an exploratory subgroup because clinical, radiographic, quantitative culture, and VAP data were unavailable; therefore, airway culture positivity could not reliably distinguish lower respiratory infection from colonization.

Both bacterial and fungal isolates were included when describing the microbiological spectrum. Antibacterial susceptibility analyses were restricted to the predominant bacterial organisms. Fungal isolates were summarized descriptively and were not included in antibacterial susceptibility analyses.

### 2.3. Microbiological Procedures and AST

Cultures and AST were performed as part of the laboratory′s routine diagnostic workflow. For bacterial culture, specimens were inoculated onto blood agar, MacConkey agar, and chocolate agar as appropriate to specimen type and routine laboratory practice. Cultures were generally incubated at 35°C–37°C for approximately 24 h, with chocolate agar incubated under carbon dioxide–enriched conditions. Bacterial and fungal isolates were identified using conventional microbiological and biochemical methods according to the laboratory′s routine diagnostic procedures.

Anaerobic culture was not performed systematically for all specimens. Seven of the 356 culture records had an explicit request for combined aerobic and anaerobic bacterial culture; six were culture‐positive and one was culture‐negative. The organisms recovered from the six positive specimens were aerobic or facultative organisms, and no obligate anaerobic organism was recorded. Accordingly, the absence of anaerobic isolates was not interpreted as evidence of absence of anaerobic infection in the source population.

Bacterial AST was performed predominantly using disk diffusion methods. MIC‐based testing was used for selected antimicrobial–organism combinations when available in the laboratory records, particularly for colistin, vancomycin, and oxacillin. Finally, *S*. *aureus* MSSA/MRSA classification was based on the verified methicillin/oxacillin resistance phenotype in the cleaned laboratory dataset.

Carbapenem susceptibility was assessed by disk diffusion. Discordant imipenem–meropenem categorical results identified during data analysis were rechecked against the original laboratory AST records and were confirmed to match the source data, excluding transcription or data‐entry error as an explanation. The specific disk potencies, original inhibition‐zone diameters, and documentation of repeat testing or MIC confirmation for these isolates were not available for retrospective review.

AST interpretation during the study period followed CLSI M100, 30th edition (2020) [11]. This interpretive standard remained the laboratory reference throughout 2022–2025. Year‐specific reinterpretation using later CLSI editions was not possible because original quantitative inhibition‐zone diameters and MIC values were not retained consistently in the analytic database. Therefore, susceptibility categories were analyzed according to the interpretations recorded by the laboratory at the time of testing.

The reporting framework was informed by CLSI M39‐A4 principles [12], including organism‐specific antimicrobial panels, explicit reporting of the number tested for each antimicrobial agent, and calculation of susceptibility percentages using agent‐specific valid denominators. Estimates based on fewer than 30 isolates were treated as preliminary descriptive observations and were not interpreted as formal cumulative antibiograms or empirical‐treatment thresholds.

For the main susceptibility analyses, results were summarized as susceptible versus nonsusceptible according to the cleaned analytic dataset; the latter category reflected originally intermediate or resistant results where these had been collapsed during database preparation. Colistin was handled separately because CLSI M100 (2020) does not provide a susceptible interpretive category for Enterobacterales or *P*. *aeruginosa*. MIC‐based colistin results were therefore summarized using resistant counts rather than susceptible percentages.

Details such as individual specimen transport times, the specific disk potencies used for retrospective carbapenem testing records, and the complete isolate‐specific biochemical identification sequence were not consistently retrievable from the laboratory information system.

### 2.4. Statistical Analysis

Data were analyzed using IBM SPSS Statistics, Version 26. Categorical variables were summarized as frequencies and percentages. Age was analyzed as a continuous variable and summarized as mean ± standard deviation; age data were complete for all 136 culture‐positive records.

Culture positivity was calculated using all 356 eligible culture records as the denominator. Subsequent microbiological analyses were performed among the 136 culture‐positive specimens. The microbiological spectrum was summarized overall and according to specimen category. Pleural‐space and deep pulmonary/lung surgical specimens were combined for the primary analysis, whereas tracheal/airway specimens were analyzed separately as an exploratory subgroup.

For each antimicrobial agent, the number tested and the number and percentage of susceptible isolates were calculated using isolates with valid AST results for that agent as the denominator. Exact two‐sided 95% confidence intervals for susceptibility proportions were calculated using the Clopper–Pearson method. Estimates based on fewer than 30 isolates were interpreted descriptively and with caution.

Source‐stratified susceptibility analyses compared primary pleural/deep surgical isolates with exploratory tracheal/airway isolates. Because of the small subgroup sizes and sparse cell counts, between‐group comparisons used two‐sided Fisher′s exact tests. These comparisons were exploratory, and no adjustment for multiple comparisons was performed.

Missing AST results represented antimicrobial agents that were not tested for a given isolate as part of the routine laboratory workflow. No imputation was performed. Testing completeness was examined descriptively by calendar year and by primary versus exploratory specimen group. Susceptibility calculations used agent‐specific tested denominators rather than treating untested isolates as susceptible or resistant.

## 3. Results

### 3.1. Study Sample and Specimen Characteristics

A total of 356 eligible thoracic and respiratory culture records were identified during the study period. Of these, 136 were culture‐positive, yielding a culture positivity rate of 38.2%, and these 136 specimens constituted the analytic cohort. The mean age of patients with culture‐positive specimens was 42.3 ± 21.5 years, and most specimens were obtained from male patients (91/136, 66.9%). Pleural‐space specimens were the most frequent specimen category (66/136, 48.5%), followed by deep pulmonary/lung surgical specimens (44/136, 32.4%) and tracheal/airway specimens (26/136, 19.1%). When grouped analytically, pleural‐space and deep pulmonary/lung surgical specimens together comprised the primary pleural/deep cohort (110/136, 80.9%), whereas tracheal/airway specimens comprised the exploratory airway cohort (26/136, 19.1%) (Table 1) Figure 1.

**Figure 1:**
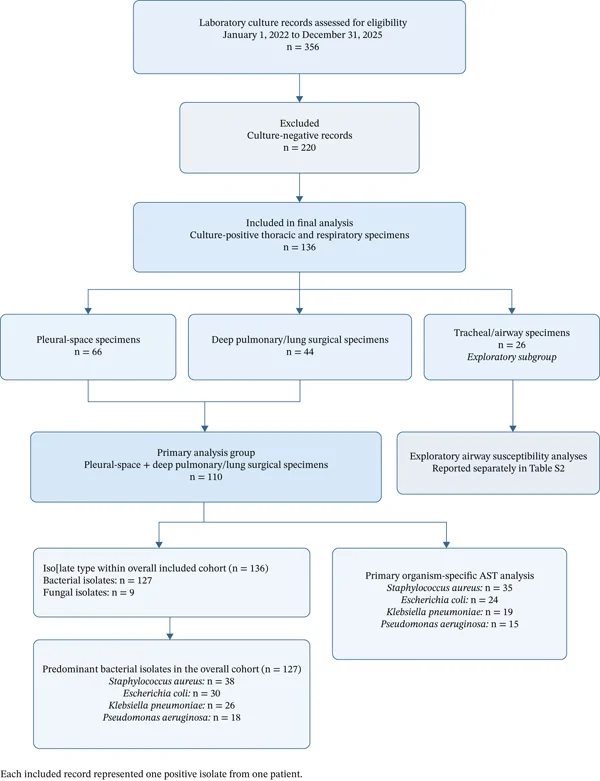
Study flow of thoracic and respiratory culture records included in the analysis. Laboratory culture records from January 1, 2022, to December 31, 2025, were extracted from the laboratory information system in February 2026. Of 356 eligible records, 220 were culture‐negative and excluded, leaving 136 culture‐positive specimens for analysis. Pleural‐space (*n* = 66) and deep pulmonary/lung surgical specimens (*n* = 44) were combined as the primary pleural/deep analysis group (*n* = 110), whereas tracheal/airway specimens (*n* = 26) were analyzed separately as an exploratory subgroup. Each included record represented one positive isolate from one patient.

**Table 1:** Demographic, specimen, and microbiological characteristics of culture‐positive thoracic specimens (*N* = 136).

Characteristic	Category	Value
Demographic characteristics		
Age, years	Mean ± SD	42.3 ± 21.5
Sex	Female	45 (33.1)
	Male	91 (66.9)
Specimen characteristics		
Specimen category	Pleural‐space specimens	66 (48.5)
	Deep pulmonary/lung surgical specimens	44 (32.4)
	Tracheal/airway specimens	26 (19.1)
Year of specimen submission		
Order year	2022	34 (25.0)
	2023	35 (25.7)
	2024	51 (37.5)
	2025	16 (11.8)
Microbiological profile		
Isolate type	Bacterial isolates	127 (93.4)
	Fungal isolates	9 (6.6)
Most frequent bacterial isolates (*N* = 127)		
	*Staphylococcus aureus*	38 (29.9)
	*Escherichia coli*	30 (23.6)
	*Klebsiella pneumoniae*	26 (20.5)
	*Pseudomonas aeruginosa*	18 (14.2)
	Other bacterial isolates ^∗^	15 (11.8)
Fungal isolates (*N* = 9)		
	*Aspergillus fumigatus*	6 (66.7)
	*Candida albicans*	3 (33.3)

*Note:* Data are presented as *n* (%) unless otherwise indicated. Age was summarized as mean ± SD. Percentages for demographic, specimen, yearly, and isolate‐type variables were calculated using all culture‐positive specimens as the denominator (*N* = 136). Percentages for bacterial and fungal spectra were calculated using bacterial isolates (*N* = 127) and fungal isolates (*N* = 9), respectively. Pleural‐space and deep pulmonary/lung surgical specimens together constituted the primary analysis group (*n* = 110, 80.9%), whereas tracheal/airway specimens (*n* = 26, 19.1%) were analyzed as an exploratory subgroup. Overall, *Staphylococcus aureus* comprised 35 MSSA and 3 MRSA isolates; among the primary pleural/deep surgical subgroup analyzed in Table 3, 32 were MSSA and 3 were MRSA.

^∗^Other bacterial isolates included *Streptococcus pyogenes* (*n* = 5), *Streptococcus pneumoniae* (*n* = 4), *Acinetobacter baumannii* (*n* = 2), *Enterobacter* spp. (*n* = 2), *Bacillus cereus* (*n* = 1), and *Proteus mirabilis* (*n* = 1).

### 3.2. Microbiological Spectrum

Bacterial isolates accounted for 127/136 culture‐positive specimens (93.4%), whereas fungal isolates accounted for 9/136 (6.6%). Among bacterial isolates, the most frequent organisms were *Staphylococcus aureus* (38/127, 29.9%), *E*. *coli* (30/127, 23.6%), *K*. *pneumoniae* (26/127, 20.5%), and *P*. *aeruginosa* (18/127, 14.2%). The *S*. *aureus* isolates comprised 35 methicillin‐susceptible *Staphylococcus aureus* (MSSA) and 3 methicillin‐resistant *Staphylococcus aureus* (MRSA). Among fungal isolates, *A*. *fumigatus* was most common (6/9, 66.7%), followed by *Candida albicans* (3/9, 33.3%) (Table 1).

When stratified by specimen category, bacterial isolates predominated across pleural‐space, deep pulmonary/lung surgical, and tracheal/airway specimens. Male patients represented the majority in all specimen groups. *S*. *aureus* was the most frequent isolate in pleural‐space and deep pulmonary/lung surgical specimens, whereas *K*. *pneumoniae* was the most frequent organism among tracheal/airway specimens. Fungal isolates were proportionally most common in the deep pulmonary/lung surgical group (Table 2).

**Table 2:** Distribution of isolated microorganisms and patient characteristics by specimen category, with primary pleural/deep and exploratory airway analyses (*N* = 136).

Characteristic/microorganism	Pleural‐space specimens (*N* = 66)	Deep pulmonary/lung surgical specimens (*N* = 44)	Primary pleural/deep surgical specimens (*N* = 110)	Exploratory tracheal/airway specimens (*N* = 26)	Total (*N* = 136)
Patient characteristics					
Age, years	39.6 ± 18.6	43.1 ± 22.6	41.0 ± 20.3	47.7 ± 25.9	42.3 ± 21.5
Sex					
Female	21 (31.8)	13 (29.5)	34 (30.9)	11 (42.3)	45 (33.1)
Male	45 (68.2)	31 (70.5)	76 (69.1)	15 (57.7)	91 (66.9)
Microorganism category					
Bacterial isolates	64 (97.0)	39 (88.6)	103 (93.6)	24 (92.3)	127 (93.4)
Fungal isolates	2 (3.0)	5 (11.4)	7 (6.4)	2 (7.7)	9 (6.6)
Most frequent bacterial microorganisms					
*Staphylococcus aureus*	20 (30.3)	15 (34.1)	35 (31.8)	3 (11.5)	38 (27.9)
*Escherichia coli*	18 (27.3)	6 (13.6)	24 (21.8)	6 (23.1)	30 (22.1)
*Klebsiella pneumoniae*	9 (13.6)	10 (22.7)	19 (17.3)	7 (26.9)	26 (19.1)
*Pseudomonas aeruginosa*	9 (13.6)	6 (13.6)	15 (13.6)	3 (11.5)	18 (13.2)
Other bacterial isolates	8 (12.1)	2 (4.5)	10 (9.1)	5 (19.2)	15 (11.0)
Fungal microorganisms					
*Aspergillus fumigatus*	2 (3.0)	4 (9.1)	6 (5.5)	0 (0.0)	6 (4.4)
*Candida albicans*	0 (0.0)	1 (2.3)	1 (0.9)	2 (7.7)	3 (2.2)

*Note:* Data are presented as *n* (%) unless otherwise indicated. Age is presented as mean ± SD. Percentages were calculated within each respective column. Pleural‐space and deep pulmonary/lung surgical specimens were combined as the primary deep/pleural analysis group. Tracheal/airway specimens were analyzed separately as an exploratory subgroup because clinical, radiographic, quantitative‐culture, and ventilator‐associated pneumonia data were unavailable; therefore, airway isolates could not be reliably distinguished as representing lower respiratory infection versus colonization. *Staphylococcus aureus* included methicillin‐susceptible *Staphylococcus aureus* (MSSA) (*n* = 35) and methicillin‐resistant *Staphylococcus* aureus (MRSA) (*n* = 3). Other bacterial isolates included *Streptococcus pyogenes* (*n* = 5), *Streptococcus pneumoniae* (*n* = 4), *Acinetobacter baumannii* (*n* = 2), *Enterobacter* spp. (*n* = 2), *Bacillus cereus* (*n* = 1), and *Proteus mirabilis* (*n* = 1).

### 3.3. Antimicrobial Susceptibility of *S*. *aureus* in the Primary Pleural/Deep Cohort

In the primary pleural/deep cohort, 35 *S*. *aureus* isolates were available for analysis, of which 32/35 (91.4%) were methicillin‐susceptible, corresponding to MSSA, and 3/35 (8.6%) were MRSA. Susceptibility was highest to vancomycin (35/35, 100.0%) and linezolid (33/35, 94.3%). Relatively higher susceptibility was also observed for levofloxacin (23/31, 74.2%), minocycline (25/35, 71.4%), and gentamicin (21/30, 70.0%). In contrast, lower susceptibility was observed for ciprofloxacin (17/31, 54.8%), azithromycin (7/31, 22.6%), and trimethoprim‐sulfamethoxazole (4/31, 12.9%) (Table 3).

**Table 3:** Antimicrobial susceptibility profile of *Staphylococcus aureus* isolates from primary pleural/deep surgical specimens (*N* = 35).

Antimicrobial class	Antimicrobial agent	*N* tested	Susceptible, *n*(%)	Exact 95% CI	MSSA, S/N	MRSA, S/N
Antistaphylococcal penicillins	Methicillin (oxacillin resistance phenotype)	35	32 (91.4)	76.9–98.2	32/32	0/3
Macrolides	Azithromycin	31	7 (22.6)	9.6–41.1	6/28	1/3
Aminoglycosides	Gentamicin	30	21 (70.0)	50.6–85.3	21/28	0/2
Fluoroquinolones	Ciprofloxacin	31	17 (54.8)	36.0–72.7	17/29	0/2
	Levofloxacin	31	23 (74.2)	55.4–88.1	23/29	0/2
Tetracyclines	Minocycline	35	25 (71.4)	53.7–85.4	24/32	1/3
Folate pathway inhibitors	Trimethoprim‐sulfamethoxazole	31	4 (12.9)	3.6–29.8	4/29	0/2
Oxazolidinones	Linezolid	35	33 (94.3)	80.8–99.3	30/32	3/3
Glycopeptides	Vancomycin	35	35 (100.0)	90.0–100.0	32/32	3/3

*Note:* Data are presented as susceptible isolates, *n* (%), calculated among isolates with valid susceptibility results for each antimicrobial agent. Exact two‐sided 95% confidence intervals were calculated using the Clopper–Pearson binomial method. The primary pleural/deep surgical *S. aureus* cohort comprised 32 MSSA and 3 MRSA isolates. MSSA and MRSA subgroup columns are presented as raw susceptible/tested counts because of the very small MRSA subgroup. Missing susceptibility results reflect variation in the routine antimicrobial testing panel and material availability during the study period. The methicillin row reflects the verified methicillin/oxacillin resistance phenotype used for final MSSA/MRSA classification in the cleaned dataset. Exploratory tracheal/airway isolates were excluded from this primary susceptibility table and are presented separately in Table S2.

Abbreviations: CI, confidence interval; MRSA, methicillin‐resistant *Staphylococcus aureus*; MSSA, methicillin‐susceptible *Staphylococcus aureus*; *N*, number of isolates tested; S/N, susceptible/tested.

### 3.4. Antimicrobial Susceptibility of *E*. *coli* in the Primary Pleural/Deep Cohort

Among the 24 primary pleural/deep *E*. *coli* isolates, complete susceptibility was observed to amikacin (24/24, 100.0%) and meropenem (24/24, 100.0%), whereas imipenem susceptibility was also high (20/24, 83.3%). Moderate susceptibility proportions were observed for piperacillin‐tazobactam (13/24, 54.2%) and levofloxacin (11/24, 45.8%), whereas gentamicin susceptibility was lower (10/24, 41.7%). Susceptibility to tobramycin (6/23, 26.1%), ciprofloxacin (4/24, 16.7%), trimethoprim‐sulfamethoxazole (3/24, 12.5%), ceftriaxone (2/23, 8.7%), and cefepime (2/24, 8.3%) was limited. MIC‐based colistin testing showed no resistant *E*. *coli* isolates (0/24; *R* *%* = 0.0*%*) (Table 4).

**Table 4:** Antimicrobial susceptibility profile of *Escherichia coli* isolates from primary pleural/deep surgical specimens (*N* = 24).

Antimicrobial class	Antimicrobial agent	*N* tested	Susceptible, *n*(%)	Exact 95% CI
Aminoglycosides	Amikacin	24	24 (100.0)	85.8–100.0
	Gentamicin	24	10 (41.7)	22.1–63.4
	Tobramycin	23	6 (26.1)	10.2–48.4
Beta‐lactam/beta‐lactamase inhibitor combinations	Piperacillin‐tazobactam	24	13 (54.2)	32.8–74.4
Cephalosporins	Cefepime	24	2 (8.3)	1.0–27.0
	Ceftriaxone	23	2 (8.7)	1.1–28.0
Fluoroquinolones	Ciprofloxacin	24	4 (16.7)	4.7–37.4
	Levofloxacin	24	11 (45.8)	25.6–67.2
Carbapenems	Imipenem	24	20 (83.3)	62.6–95.3
	Meropenem	24	24 (100.0)	85.8–100.0
Folate pathway inhibitors	Trimethoprim‐sulfamethoxazole	24	3 (12.5)	2.7–32.4

*Note:* Data are presented as susceptible isolates, *n* (%), calculated among isolates with valid susceptibility results for each antimicrobial agent. Exact two‐sided 95% confidence intervals were calculated using the Clopper–Pearson binomial method. Because only 24 *E*. *coli* isolates were available in the primary pleural/deep surgical subgroup, these estimates are preliminary descriptive observations and should not be interpreted as a formal cumulative antibiogram or empirical treatment threshold. Agent‐specific denominators varied because of differences in the routine antimicrobial testing panel and intermittent material availability during the study period. Exploratory tracheal/airway isolates are reported separately in Table S2. Colistin was summarized separately because CLSI M100 (2020) provides intermediate and resistant, but no susceptible interpretive category for Enterobacterales. MIC‐based colistin testing showed 0/24 resistant *E*. *coli* isolates in the primary pleural/deep surgical subgroup.

Abbreviations: CI, confidence interval; *N*, number of isolates tested.

### 3.5. Antimicrobial Susceptibility of *K*. *pneumoniae* in the Primary Pleural/Deep Cohort

Among the 19 primary pleural/deep *K*. *pneumoniae* isolates, complete susceptibility was observed to amikacin (19/19, 100.0%) and meropenem (17/17, 100.0%), whereas susceptibility to imipenem was also high (17/19, 89.5%). Piperacillin‐tazobactam demonstrated substantial in vitro activity (16/18, 88.9%), and levofloxacin showed moderate‐to‐high susceptibility (14/19, 73.7%). Gentamicin susceptibility was 52.6% (10/19). Lower susceptibility was observed for tobramycin (5/18, 27.8%), ceftriaxone (4/19, 21.1%), ampicillin‐sulbactam (3/18, 16.7%), ciprofloxacin (3/19, 15.8%), cefepime (2/18, 11.1%), and trimethoprim‐sulfamethoxazole (1/19, 5.3%). MIC‐based colistin testing showed no resistant *K*. *pneumoniae* isolates (0/19; *R* *%* = 0.0*%*). Because fewer than 30 isolates were available, these susceptibility estimates should be interpreted descriptively and with caution (Table 5).

**Table 5:** Antimicrobial susceptibility profile of *Klebsiella pneumoniae* isolates from primary pleural/deep surgical specimens (*N* = 19).

Antimicrobial class	Antimicrobial agent	*N* tested	Susceptible, *n*(%)	Exact 95% CI
Aminoglycosides	Amikacin	19	19 (100.0)	82.4–100.0
	Gentamicin	19	10 (52.6)	28.9–75.6
	Tobramycin	18	5 (27.8)	9.7–53.5
Beta‐lactam/beta‐lactamase inhibitor combinations	Ampicillin‐sulbactam	18	3 (16.7)	3.6–41.4
	Piperacillin‐tazobactam	18	16 (88.9)	65.3–98.6
Cephalosporins	Cefepime	18	2 (11.1)	1.4–34.7
	Ceftriaxone	19	4 (21.1)	6.1–45.6
Carbapenems	Imipenem	19	17 (89.5)	66.9–98.7
	Meropenem	17	17 (100.0)	80.5–100.0
Fluoroquinolones	Ciprofloxacin	19	3 (15.8)	3.4–39.6
	Levofloxacin	19	14 (73.7)	48.8–90.9
Folate pathway inhibitors	Trimethoprim‐sulfamethoxazole	19	1 (5.3)	0.1–26.0

*Note:* Data are presented as susceptible isolates, *n* (%), calculated among isolates with valid susceptibility results for each antimicrobial agent. Exact two‐sided 95% confidence intervals were calculated using the Clopper–Pearson binomial method. Because only 19 *K*. *pneumoniae* isolates were available in the primary pleural/deep surgical subgroup, these estimates are preliminary descriptive observations and should not be interpreted as a formal cumulative antibiogram or empirical treatment threshold. Agent‐specific denominators varied because of differences in the routine antimicrobial testing panel and intermittent material availability during the study period. Exploratory tracheal/airway isolates are reported separately in Table S2. Colistin was summarized separately because CLSI M100 (2020) does not provide a susceptible interpretive category for Enterobacterales. MIC‐based colistin testing showed 0/19 resistant K. pneumoniae isolates in the primary pleural/deep surgical subgroup.

Abbreviations: CI, confidence interval; *N*, number of isolates tested.

### 3.6. Supplementary Susceptibility Findings for *P*. *aeruginosa*

Because only 15 primary pleural/deep *P*. *aeruginosa* isolates were available, organism‐specific susceptibility estimates were presented separately in Table S1. The highest susceptibility was observed for amikacin and meropenem (14/15 each, 93.3%), followed by imipenem (12/15, 80.0%) and piperacillin‐tazobactam (11/15, 73.3%). Lower susceptibility was observed for gentamicin (6/15, 40.0%), levofloxacin (5/14, 35.7%), tobramycin (4/14, 28.6%), ciprofloxacin (4/15, 26.7%), and cefepime (2/15, 13.3%). No colistin‐resistant isolate was identified (0/15; *R* *%* = 0.0*%*) (Table S1).

### 3.7. Exploratory Source‐Stratified Susceptibility Analysis

Exploratory comparisons between the primary pleural/deep cohort and the tracheal/airway cohort are presented in Table S2. For *S*. *aureus*, *E*. *coli*, and *P*. *aeruginosa*, susceptibility patterns were broadly similar between specimen groups, although some apparent differences were noted in small airway subgroups. The most notable divergence was observed for *K*. *pneumoniae*, for which primary pleural/deep isolates showed substantially higher susceptibility than airway isolates to piperacillin‐tazobactam (88.9% vs. 14.3%, *p* = 0.001), levofloxacin (73.7% vs. 0.0%, *p* = 0.001), and imipenem (89.5% vs. 28.6%, *p* = 0.006) (Table S2).

### 3.8. Completeness of AST

The completeness of AST varied across organism–antimicrobial combinations and calendar years and is summarized in Table S3. Testing completeness was generally high for *E*. *coli* and *P*. *aeruginosa*. In contrast, more notable incompleteness was observed for selected *S*. *aureus* agents in 2025 and for several *K*. *pneumoniae* agents in 2022 and 2025. These findings support the use of antimicrobial‐specific denominators in all susceptibility summaries (Table S3).

## 4. Discussion

### 4.1. Gram‐Positive Isolates and Anti‐Staphylococcal Susceptibility

*S*. *aureus* was the most frequently recovered organism in the culture‐positive cohort and remained prominent within the primary pleural/deep analysis group. Comparable prominence of *S*. *aureus* has been reported in pleural/sterile‐fluid datasets from India and in Lebanese wound and surgical‐site infections [13–15]. However, this pattern differs from some community‐acquired pleural infection cohorts, particularly from Europe, where nonpneumococcal streptococci predominated and gram‐negative organisms were less frequent [3]. These differences underscore the heterogeneity of organism distributions across specimen types and clinical settings and caution against direct comparison between community‐acquired pleural cohorts and referral‐laboratory thoracic specimens.

In the primary pleural/deep *S*. *aureus* analysis, 3 of 35 isolates were MRSA, a low proportion that should be interpreted cautiously given the small number of resistant isolates. Regional studies have reported substantial variation in methicillin resistance, ranging from high methicillin resistance and multidrug resistance among Sudanese *Staphylococcus* spp. isolates to notable MRSA proportions in Indian sterile‐fluid and Nigerian empyema cohorts [2, 13, 16]. Lebanese surveillance reported a substantial MRSA burden while retaining high activity of key anti‐staphylococcal agents [15]. In our cohort, vancomycin and linezolid retained high in vitro activity against *S*. *aureus*, whereas trimethoprim‐sulfamethoxazole and macrolides showed poor activity and fluoroquinolones showed only moderate susceptibility. In the Syrian context, where substantial nonprescription and pharmacist‐directed antibiotic use has been documented, the low in vitro activity of several commonly used agents reinforces the value of isolate‐specific AST for interpreting culture‐positive thoracic specimens [9].

### 4.2. Gram‐Negative Organisms and Regional AMR Context

The substantial contribution of *E*. *coli*, *K*. *pneumoniae*, and *P*. *aeruginosa* places our findings within a growing body of literature showing that complicated thoracic infections are not uniformly dominated by streptococci and anaerobes. Nigerian and Pakistani empyema studies reported prominent gram‐negative involvement, particularly *Klebsiella*, *Pseudomonas*, and *E*. *coli*, whereas Taiwanese lung abscess data challenged the classical anaerobic paradigm by identifying *K*. *pneumoniae* as an important pathogen [2, 5, 17]. These findings contrast with European community‐acquired pleural infection data, where gram‐positive cocci were more prominent. These findings emphasize that microbiological patterns from community‐acquired pleural cohorts should not be directly extrapolated to heterogeneous referral‐laboratory thoracic specimens [3].

Broader regional data further support this interpretation. Studies from Morocco, India, Uganda, Kuwait, Saudi Arabia, Jordan, Somalia, Iran, and Romania repeatedly identify *E*. *coli*, *K*. *pneumoniae*, *P*. *aeruginosa*, and *Acinetobacter* spp. among important organisms in nosocomial, sterile‐site, ICU, chest, and respiratory‐associated datasets [1, 4, 13, 14, 18–23]. Although heterogeneous in design and specimen source, these studies consistently document an important gram‐negative burden in hospital, ICU, respiratory, and sterile‐site datasets. Our findings therefore align with regional hospital‐based and respiratory/sterile‐site AMR datasets, while remaining distinct from any single clinical syndrome.

In the primary pleural/deep analysis, amikacin and meropenem showed comparatively high in vitro activity among the dominant gram‐negative isolates; however, organism‐specific denominators were small (*E*. *coli*, *n* = 24; *K*. *pneumoniae*, *n* = 19; *P*. *aeruginosa*, *n* = 15), and these estimates should be interpreted as preliminary descriptive observations rather than formal cumulative antibiograms or empirical‐treatment thresholds. This general pattern is consistent with Nigerian empyema data showing high resistance to ceftriaxone and amoxicillin‐clavulanate, with comparatively lower resistance to imipenem, piperacillin‐tazobactam, amikacin, and levofloxacin [2]. Saudi ICU chest infection data also reported substantial resistance among *Klebsiella*, *Pseudomonas*, and *Acinetobacter* isolates [4]. A Saudi national *P*. *aeruginosa* surveillance study reported amikacin as the agent with the highest susceptibility (76.8%), whereas meropenem susceptibility was 52% [24]. Kuwaiti data similarly demonstrated substantial multidrug resistance among gram‐negative isolates, particularly in respiratory specimens [22].

The apparent difference between imipenem and meropenem results also warrants caution. Meropenem susceptibility was numerically higher than imipenem susceptibility in the primary analyses of *E*. *coli*, *K*. *pneumoniae*, and *P*. *aeruginosa*. Because the analytic database did not retain the original quantitative MIC or inhibition‐zone measurements for these isolates, the discrepant categorical results could not be reinterpreted or mechanistically resolved. Accordingly, these findings should not be taken as evidence of intrinsic superiority of one carbapenem or of a specific resistance mechanism.

Exploratory source‐stratified analyses showed no consistent source‐related susceptibility differences for *S*. *aureus*, *E*. *coli*, or *P*. *aeruginosa*. In contrast, *K*. *pneumoniae* airway isolates showed lower susceptibility than primary pleural/deep isolates to piperacillin‐tazobactam (14.3% vs. 88.9%; Fisher′s exact *p* = 0.001), levofloxacin (0% vs. 73.7%; *p* = 0.001), and imipenem (28.6% vs. 89.5%; *p* = 0.006). These findings are hypothesis‐generating only because the airway *K*. *pneumoniae* subgroup contained seven isolates and no adjustment for multiple comparisons was performed.

Resistance mechanisms documented in regional studies provide useful external context but cannot be inferred from our phenotypic susceptibility data. Patel et al. [14] identified ESBL, metallo‐beta‐lactamase, and AmpC phenotypes among gram‐negative sterile body–fluid isolates, whereas Rahimzadeh et al. [23] detected multiple resistance‐associated genetic determinants among MDR gram‐negative pneumonia isolates; a Jordanian HAP/VAP study likewise documented several *β*‐lactam and quinolone resistance determinants [1]. However, our study did not perform phenotypic confirmation or molecular testing of resistance mechanisms. Therefore, the low susceptibility to cephalosporins and fluoroquinolones in our isolates should be interpreted as a phenotypic susceptibility pattern only and cannot be attributed to ESBLs, carbapenemases, or specific quinolone‐resistance mechanisms. Notably, low cephalosporin activity has also been reported in another Syrian laboratory‐based study, including pooled Enterobacterales and several tested oral cephalosporins [9]. That study discussed these findings in the context of substantial community antibiotic exposure in Syria, including nonprescription access, pharmacist‐directed use, incomplete treatment courses, and frequent consumption of broad‐spectrum cephalosporins, all of which may plausibly contribute to antimicrobial selection pressure [9]. Nevertheless, neither the present study nor the prior Syrian study can establish a direct causal relationship between individual antibiotic exposure and the observed resistance patterns.

MIC‐based colistin testing showed no resistant isolates among the dominant gram‐negative organisms. However, colistin was summarized separately because CLSI M100 2020 does not assign a susceptible category for colistin in Enterobacterales or *P*. *aeruginosa* [11]. Accordingly, these results should not be interpreted as “colistin susceptibility.”

### 4.3. Interpretation and Implications

The findings should be interpreted in the context of a referral microbiology laboratory receiving specimens from multiple physicians, hospitals, and surgical services rather than a defined hospital catchment population. Regional hospital and ICU studies document substantial gram‐negative and antimicrobial‐resistant burdens in respiratory, pleural, and other clinical specimens [1–4, 18–24]. Syrian data summarized in a prior local study also describe substantial nonprescription antibiotic access, pharmacist‐directed use, incomplete courses, and high Watch‐group consumption [9]. These external observations provide context for the relevance of local resistance surveillance but do not establish prior antimicrobial exposure or healthcare‐associated acquisition for individual patients in our cohort.

For pleural or deep thoracic specimens, complete microbiological work‐up, including anaerobic culture when clinically indicated, remains important because anaerobic organisms may be missed when such culture is not systematically performed [2, 17]. The present susceptibility estimates are best viewed as local laboratory‐based signals supporting culture‐guided interpretation and antimicrobial‐resistance surveillance. Because the principal gram‐negative organism‐specific denominators were below 30 and clinical outcome data were unavailable, these data should not be used alone to define empirical treatment regimens.

### 4.4. Limitations

This study has several limitations. First, it was retrospective and laboratory‐based, without linked clinical data on prior antibiotic exposure, radiological findings, operative findings, disease severity, source‐control procedures, treatment regimens, or clinical outcomes. Second, tracheal and airway isolates may represent infection or colonization, and clinical correlation was not available to distinguish these entities. Third, anaerobic culture was explicitly requested for only 7 of 356 specimens; no obligate anaerobic isolate was recovered, so the study cannot estimate the anaerobic burden and the absence of anaerobic isolates should not be interpreted as true absence. Fourth, the primary susceptibility analyses for *E*. *coli* (*n* = 24), *K*. *pneumoniae* (*n* = 19), and *P*. *aeruginosa* (*n* = 15) were based on fewer than 30 isolates, resulting in substantial statistical uncertainty; these estimates were therefore presented as descriptive observations rather than formal cumulative antibiograms. Fifth, fungal isolates were few and were therefore interpreted descriptively. AST was performed predominantly using routine disk diffusion methods, whereas MIC‐based testing was available only for selected antimicrobial–organism combinations as recorded in the laboratory database. AST completeness varied by antimicrobial agent and calendar year as part of routine laboratory workflow and intermittent material availability; missing AST results were not imputed and cannot be assumed to be completely random. Quantitative MIC values and inhibition‐zone diameters were not consistently retained in the analytic database, precluding reinterpretation under later CLSI editions and limiting investigation of discordant carbapenem results. Finally, because the data came from a private referral laboratory receiving specimens from multiple external sources, the study population may not represent the microbiological spectrum of a defined hospital catchment population. Despite these limitations, the study provides locally relevant laboratory‐based data on the microbiological spectrum and in vitro susceptibility patterns of culture‐positive thoracic and respiratory specimens in Syria.

## 5. Conclusion

Culture‐positive thoracic and respiratory specimens processed in this Syrian referral laboratory showed a microbiological spectrum dominated by *S*. *aureus* and clinically important gram‐negative bacilli, particularly *E*. *coli*, *K*. *pneumoniae*, and *P*. *aeruginosa*. In the primary pleural/deep analysis, vancomycin and linezolid showed high in vitro activity against *S*. *aureus*, whereas amikacin and meropenem showed comparatively high activity against the dominant gram‐negative isolates; no colistin‐resistant isolates were identified. In contrast, several cephalosporins, fluoroquinolones, and trimethoprim‐sulfamethoxazole showed lower in vitro susceptibility. Given the small organism‐specific denominators and the exploratory nature of airway analyses, these findings should be interpreted as local laboratory‐based susceptibility signals that support organism‐specific AST interpretation and antimicrobial‐resistance surveillance rather than as formal empirical‐treatment recommendations.

## Author Contributions

**Mohammad Alaa Aldakak** conceived the study, designed the methodology, performed data cleaning and statistical analysis, prepared the tables and figures, interpreted the results, and drafted the initial manuscript. **Alaa Senjab** and **Hussein Aljbawi** contributed to study design, literature review, interpretation of findings, and manuscript drafting and revision. **Ibrahim Alkhatib** and **Adnan Alkhatib** contributed to data acquisition from the laboratory information system, microbiological data verification, interpretation of culture and antimicrobial susceptibility results, and critical revision of the manuscript. **Bassam Darwish** supervised the study, contributed to the interpretation of findings, provided scientific and clinical guidance, and critically revised the manuscript for important intellectual content.

## Funding

No funding was received for this manuscript.

## Disclosure

All authors reviewed and approved the final manuscript and agreed to be accountable for all aspects of the work, including the accuracy and integrity of the data and analyses.

## Ethics Statement

The study protocol was approved by the Ethics Committee of the Syrian Private University (SPU) (Approval No. MD‐30032026‐140). This was a retrospective laboratory‐based study using deidentified data extracted from the laboratory information system; therefore, the requirement for informed consent to participate was waived by the Ethics Committee. The study was conducted in accordance with the Declaration of Helsinki.

## Consent

It is not applicable. The manuscript contains no identifiable personal data from any participant.

## Conflicts of Interest

The authors declare no conflicts of interest.

## Supporting Information

Additional supporting information can be found online in the Supporting Information section.

## Supporting information


**Supporting Information 1** Table S1: Antimicrobial susceptibility profile of *Pseudomonas aeruginosa* isolates from primary pleural/deep surgical specimens (*N* = 15).

**Supporting Information 2** Table S2: Source‐stratified antimicrobial susceptibility of predominant bacterial isolates.

**Supporting Information 3** Table S3: Antimicrobial susceptibility testing completeness by calendar year among primary pleural/deep surgical isolates.

**Supporting Information 4**  

## Data Availability

The data that support the findings of this study are available from the corresponding author upon reasonable request.
