# Supplementary material for: Microbiological Spectrum and Antimicrobial Susceptibility Patterns of Culture‐Positive Thoracic and Respiratory Specimens in Syria: A Retrospective Laboratory‐Based Study
Source: Int J Microbiol. 2026 Oct 2;2026:5705248. doi: 10.1155/ijm/5705248 (PMC13633434; doi:10.1155/ijm/5705248)
Supplement: Supplementary file 1 [file IJM-2026-5705248-s001.docx]

**Supplementary Table S1. Antimicrobial susceptibility profile of *Pseudomonas aeruginosa* isolates from primary pleural/deep surgical specimens (N = 15).**

| **Antimicrobial class** | **Antimicrobial agent** | **N tested** | **Susceptible, n (%)** | **Exact 95% CI** |
| --- | --- | --- | --- | --- |
| Aminoglycosides | Amikacin | 15 | 14 (93.3) | 68.1-99.8 |
|  | Gentamicin | 15 | 6 (40.0) | 16.3-67.7 |
|  | Tobramycin | 14 | 4 (28.6) | 8.4-58.1 |
| Beta-lactam/beta-lactamase inhibitor combinations | Piperacillin-tazobactam | 15 | 11 (73.3) | 44.9-92.2 |
| Antipseudomonal cephalosporins | Cefepime | 15 | 2 (13.3) | 1.7-40.5 |
| Fluoroquinolones | Ciprofloxacin | 15 | 4 (26.7) | 7.8-55.1 |
|  | Levofloxacin | 14 | 5 (35.7) | 12.8-64.9 |
| Carbapenems | Imipenem | 15 | 12 (80.0) | 51.9-95.7 |
|  | Meropenem | 15 | 14 (93.3) | 68.1-99.8 |

Abbreviations: CI, confidence interval; N, number of isolates tested.

• Data are presented as susceptible isolates, n (%), calculated among isolates with valid susceptibility results for each antimicrobial agent.

• Exact two-sided 95% confidence intervals were calculated using the Clopper-Pearson binomial method.

• Because only 15 *P. aeruginosa* isolates were available in the primary pleural/deep surgical subgroup, all susceptibility estimates are preliminary descriptive observations and should not be interpreted as a formal cumulative antibiogram or empirical-treatment threshold.

• Colistin was summarized separately because CLSI M100 (2020) does not provide a susceptible interpretive category for *P. aeruginosa*. MIC-based colistin testing showed 0/15 resistant isolates in the primary pleural/deep surgical subgroup.

• Exploratory tracheal/airway *P. aeruginosa* isolates are presented separately in **Supplementary Table S2**.
