# Supplementary material for: Microbiological Spectrum and Antimicrobial Susceptibility Patterns of Culture‐Positive Thoracic and Respiratory Specimens in Syria: A Retrospective Laboratory‐Based Study
Source: Int J Microbiol. 2026 Oct 2;2026:5705248. doi: 10.1155/ijm/5705248 (PMC13633434; doi:10.1155/ijm/5705248)
Supplement: Supplementary file 2 [file IJM-2026-5705248-s002.docx]

**Supplementary Table S2. Source-stratified antimicrobial susceptibility of predominant bacterial isolates**

| **Organism / antimicrobial** | **Primary pleural/deep surgical specimens Susceptible, n/N (%)** | **Exploratory tracheal/airway specimens Susceptible, n/N (%)** | **Fisher's exact p value (two-sided)** |
| --- | --- | --- | --- |
| ***Staphylococcus aureus (primary n=35; airway n=3)*** | | | |
| Methicillin phenotype | 32/35 (91.4%) | 3/3 (100.0%) | 1.000 |
| Azithromycin | 7/31 (22.6%) | 0/3 (0.0%) | 1.000 |
| Gentamicin | 21/30 (70.0%) | 2/3 (66.7%) | 1.000 |
| Ciprofloxacin | 17/31 (54.8%) | 0/3 (0.0%) | 0.227 |
| Levofloxacin | 23/31 (74.2%) | 1/3 (33.3%) | 0.201 |
| Minocycline | 25/35 (71.4%) | 2/3 (66.7%) | 1.000 |
| Trimethoprim-sulfamethoxazole | 4/31 (12.9%) | 0/3 (0.0%) | 1.000 |
| Linezolid | 33/35 (94.3%) | 3/3 (100.0%) | 1.000 |
| Vancomycin | 35/35 (100.0%) | 3/3 (100.0%) | — |
| ***Escherichia coli (primary n=24; airway n=6)*** | | | |
| Amikacin | 24/24 (100.0%) | 6/6 (100.0%) | — |
| Gentamicin | 10/24 (41.7%) | 2/6 (33.3%) | 1.000 |
| Tobramycin | 6/23 (26.1%) | 1/6 (16.7%) | 1.000 |
| Piperacillin-tazobactam | 13/24 (54.2%) | 2/5 (40.0%) | 0.651 |
| Cefepime | 2/24 (8.3%) | 0/6 (0.0%) | 1.000 |
| Ceftriaxone | 2/23 (8.7%) | 0/6 (0.0%) | 1.000 |
| Ciprofloxacin | 4/24 (16.7%) | 0/6 (0.0%) | 0.557 |
| Levofloxacin | 11/24 (45.8%) | 0/6 (0.0%) | 0.061 |
| Imipenem | 20/24 (83.3%) | 4/6 (66.7%) | 0.571 |
| Meropenem | 24/24 (100.0%) | 6/6 (100.0%) | — |
| Trimethoprim-sulfamethoxazole | 3/24 (12.5%) | 0/6 (0.0%) | 1.000 |
| ***Klebsiella pneumoniae (primary n=19; airway n=7)*** | | | |
| Amikacin | 19/19 (100.0%) | 7/7 (100.0%) | — |
| Gentamicin | 10/19 (52.6%) | 1/7 (14.3%) | 0.178 |
| Tobramycin | 5/18 (27.8%) | 2/7 (28.6%) | 1.000 |
| Ampicillin-sulbactam | 3/18 (16.7%) | 0/7 (0.0%) | 0.534 |
| Piperacillin-tazobactam | 16/18 (88.9%) | 1/7 (14.3%) | **0.001** |
| Cefepime | 2/18 (11.1%) | 0/7 (0.0%) | 1.000 |
| Ceftriaxone | 4/19 (21.1%) | 0/7 (0.0%) | 0.546 |
| Ciprofloxacin | 3/19 (15.8%) | 0/7 (0.0%) | 0.540 |
| Levofloxacin | 14/19 (73.7%) | 0/7 (0.0%) | **0.001** |
| Imipenem | 17/19 (89.5%) | 2/7 (28.6%) | **0.006** |
| Meropenem | 17/17 (100.0%) | 7/7 (100.0%) | — |
| Trimethoprim-sulfamethoxazole | 1/19 (5.3%) | 0/7 (0.0%) | 1.000 |
| ***Pseudomonas aeruginosa (primary n=15; airway n=3)*** | | | |
| Amikacin | 14/15 (93.3%) | 3/3 (100.0%) | 1.000 |
| Gentamicin | 6/15 (40.0%) | 1/3 (33.3%) | 1.000 |
| Tobramycin | 4/14 (28.6%) | 0/3 (0.0%) | 0.541 |
| Piperacillin-tazobactam | 11/15 (73.3%) | 2/3 (66.7%) | 1.000 |
| Cefepime | 2/15 (13.3%) | 0/3 (0.0%) | 1.000 |
| Ciprofloxacin | 4/15 (26.7%) | 0/3 (0.0%) | 1.000 |
| Levofloxacin | 5/14 (35.7%) | 0/3 (0.0%) | 0.515 |
| Imipenem | 12/15 (80.0%) | 1/3 (33.3%) | 0.172 |
| Meropenem | 14/15 (93.3%) | 3/3 (100.0%) | 1.000 |

Data are presented as susceptible isolates divided by the number tested, with the corresponding percentage in parentheses. Denominators vary by antimicrobial because the routine testing panel was not identical for every isolate.

Primary analysis includes pleural-space and deep pulmonary/lung surgical specimens. Tracheal/airway specimens are shown as an exploratory subgroup because clinical and radiographic data were unavailable to distinguish infection from colonization.

P values are from two-sided Fisher's exact tests and are exploratory; no adjustment for multiple comparisons was applied. An em dash indicates that a between-group test was not informative because susceptibility was constant across both groups.

For Pseudomonas aeruginosa, the overall susceptibility summary is reported separately in Supplementary Table S1 because of the small isolate count; it is retained here only for source-stratified exploratory comparison.

Abbreviations: TMP-SMX, trimethoprim-sulfamethoxazole.
