# Supplementary material for: Microbiological Spectrum and Antimicrobial Susceptibility Patterns of Culture‐Positive Thoracic and Respiratory Specimens in Syria: A Retrospective Laboratory‐Based Study
Source: Int J Microbiol. 2026 Oct 2;2026:5705248. doi: 10.1155/ijm/5705248 (PMC13633434; doi:10.1155/ijm/5705248)
Supplement: Supplementary file 3 [file IJM-2026-5705248-s003.docx]

**Supplementary Table S3. Antimicrobial susceptibility testing completeness by calendar year among primary pleural/deep surgical isolates.**

| **Organism** | **Antimicrobial agent** | **2022, tested/total (%)** | **2023, tested/total (%)** | **2024, tested/total (%)** | **2025, tested/total (%)** | **Overall, tested/total (%)** |
| --- | --- | --- | --- | --- | --- | --- |
| ***Staphylococcus aureus*** | | | | | | |
|  | Methicillin | 8/8 (100.0%) | 10/10 (100.0%) | 9/9 (100.0%) | 8/8 (100.0%) | 35/35 (100.0%) |
|  | Azithromycin | 6/8 (75.0%) | 9/10 (90.0%) | 9/9 (100.0%) | 7/8 (87.5%) | 31/35 (88.6%) |
|  | Gentamicin | 8/8 (100.0%) | 9/10 (90.0%) | 9/9 (100.0%) | 4/8 (50.0%) | 30/35 (85.7%) |
|  | Ciprofloxacin | 8/8 (100.0%) | 10/10 (100.0%) | 9/9 (100.0%) | 4/8 (50.0%) | 31/35 (88.6%) |
|  | Levofloxacin | 8/8 (100.0%) | 10/10 (100.0%) | 9/9 (100.0%) | 4/8 (50.0%) | 31/35 (88.6%) |
|  | Minocycline | 8/8 (100.0%) | 10/10 (100.0%) | 9/9 (100.0%) | 8/8 (100.0%) | 35/35 (100.0%) |
|  | Trimethoprim-sulfamethoxazole | 8/8 (100.0%) | 10/10 (100.0%) | 9/9 (100.0%) | 4/8 (50.0%) | 31/35 (88.6%) |
|  | Linezolid | 8/8 (100.0%) | 10/10 (100.0%) | 9/9 (100.0%) | 8/8 (100.0%) | 35/35 (100.0%) |
|  | Vancomycin | 8/8 (100.0%) | 10/10 (100.0%) | 9/9 (100.0%) | 8/8 (100.0%) | 35/35 (100.0%) |
| ***Escherichia coli*** | | | | | | |
|  | Amikacin | 8/8 (100.0%) | 6/6 (100.0%) | 7/7 (100.0%) | 3/3 (100.0%) | 24/24 (100.0%) |
|  | Gentamicin | 8/8 (100.0%) | 6/6 (100.0%) | 7/7 (100.0%) | 3/3 (100.0%) | 24/24 (100.0%) |
|  | Tobramycin | 8/8 (100.0%) | 6/6 (100.0%) | 7/7 (100.0%) | 2/3 (66.7%) | 23/24 (95.8%) |
|  | Piperacillin-tazobactam | 8/8 (100.0%) | 6/6 (100.0%) | 7/7 (100.0%) | 3/3 (100.0%) | 24/24 (100.0%) |
|  | Cefepime | 8/8 (100.0%) | 6/6 (100.0%) | 7/7 (100.0%) | 3/3 (100.0%) | 24/24 (100.0%) |
|  | Ceftriaxone | 8/8 (100.0%) | 6/6 (100.0%) | 6/7 (85.7%) | 3/3 (100.0%) | 23/24 (95.8%) |
|  | Ciprofloxacin | 8/8 (100.0%) | 6/6 (100.0%) | 7/7 (100.0%) | 3/3 (100.0%) | 24/24 (100.0%) |
|  | Levofloxacin | 8/8 (100.0%) | 6/6 (100.0%) | 7/7 (100.0%) | 3/3 (100.0%) | 24/24 (100.0%) |
|  | Imipenem | 8/8 (100.0%) | 6/6 (100.0%) | 7/7 (100.0%) | 3/3 (100.0%) | 24/24 (100.0%) |
|  | Meropenem | 8/8 (100.0%) | 6/6 (100.0%) | 7/7 (100.0%) | 3/3 (100.0%) | 24/24 (100.0%) |
|  | Trimethoprim-sulfamethoxazole | 8/8 (100.0%) | 6/6 (100.0%) | 7/7 (100.0%) | 3/3 (100.0%) | 24/24 (100.0%) |
| ***Klebsiella pneumoniae*** | | | | | | |
|  | Amikacin | 6/6 (100.0%) | 2/2 (100.0%) | 9/9 (100.0%) | 2/2 (100.0%) | 19/19 (100.0%) |
|  | Gentamicin | 6/6 (100.0%) | 2/2 (100.0%) | 9/9 (100.0%) | 2/2 (100.0%) | 19/19 (100.0%) |
|  | Tobramycin | 5/6 (83.3%) | 2/2 (100.0%) | 9/9 (100.0%) | 2/2 (100.0%) | 18/19 (94.7%) |
|  | Ampicillin-sulbactam | 6/6 (100.0%) | 2/2 (100.0%) | 9/9 (100.0%) | 1/2 (50.0%) | 18/19 (94.7%) |
|  | Piperacillin-tazobactam | 6/6 (100.0%) | 2/2 (100.0%) | 9/9 (100.0%) | 1/2 (50.0%) | 18/19 (94.7%) |
|  | Cefepime | 5/6 (83.3%) | 2/2 (100.0%) | 9/9 (100.0%) | 2/2 (100.0%) | 18/19 (94.7%) |
|  | Ceftriaxone | 6/6 (100.0%) | 2/2 (100.0%) | 9/9 (100.0%) | 2/2 (100.0%) | 19/19 (100.0%) |
|  | Ciprofloxacin | 6/6 (100.0%) | 2/2 (100.0%) | 9/9 (100.0%) | 2/2 (100.0%) | 19/19 (100.0%) |
|  | Levofloxacin | 6/6 (100.0%) | 2/2 (100.0%) | 9/9 (100.0%) | 2/2 (100.0%) | 19/19 (100.0%) |
|  | Imipenem | 6/6 (100.0%) | 2/2 (100.0%) | 9/9 (100.0%) | 2/2 (100.0%) | 19/19 (100.0%) |
|  | Meropenem | 5/6 (83.3%) | 2/2 (100.0%) | 9/9 (100.0%) | 1/2 (50.0%) | 17/19 (89.5%) |
|  | Trimethoprim-sulfamethoxazole | 6/6 (100.0%) | 2/2 (100.0%) | 9/9 (100.0%) | 2/2 (100.0%) | 19/19 (100.0%) |
| ***Pseudomonas aeruginosa*** | | | | | | |
|  | Amikacin | 4/4 (100.0%) | 5/5 (100.0%) | 5/5 (100.0%) | 1/1 (100.0%) | 15/15 (100.0%) |
|  | Gentamicin | 4/4 (100.0%) | 5/5 (100.0%) | 5/5 (100.0%) | 1/1 (100.0%) | 15/15 (100.0%) |
|  | Tobramycin | 4/4 (100.0%) | 5/5 (100.0%) | 5/5 (100.0%) | 0/1 (0.0%) | 14/15 (93.3%) |
|  | Piperacillin-tazobactam | 4/4 (100.0%) | 5/5 (100.0%) | 5/5 (100.0%) | 1/1 (100.0%) | 15/15 (100.0%) |
|  | Cefepime | 4/4 (100.0%) | 5/5 (100.0%) | 5/5 (100.0%) | 1/1 (100.0%) | 15/15 (100.0%) |
|  | Ciprofloxacin | 4/4 (100.0%) | 5/5 (100.0%) | 5/5 (100.0%) | 1/1 (100.0%) | 15/15 (100.0%) |
|  | Levofloxacin | 3/4 (75.0%) | 5/5 (100.0%) | 5/5 (100.0%) | 1/1 (100.0%) | 14/15 (93.3%) |
|  | Imipenem | 4/4 (100.0%) | 5/5 (100.0%) | 5/5 (100.0%) | 1/1 (100.0%) | 15/15 (100.0%) |
|  | Meropenem | 4/4 (100.0%) | 5/5 (100.0%) | 5/5 (100.0%) | 1/1 (100.0%) | 15/15 (100.0%) |

**Notes:** Values are number tested/number of isolates available in that organism-year stratum (% testing completeness), not susceptibility percentages. The table is restricted to the primary pleural/deep surgical analysis group. Missing AST results represent antimicrobial agents not tested for a given isolate; no imputation was performed. Yellow shading denotes strata with incomplete testing (<100%). *Year-specific completeness percentages should be interpreted cautiously because some organism-year strata were small, particularly in 2025.* Abbreviation: AST, antimicrobial susceptibility testing.
