# Supplementary material for: Microbiological Spectrum and Antimicrobial Susceptibility Patterns of Culture‐Positive Thoracic and Respiratory Specimens in Syria: A Retrospective Laboratory‐Based Study
Source: Int J Microbiol. 2026 Oct 2;2026:5705248. doi: 10.1155/ijm/5705248 (PMC13633434; doi:10.1155/ijm/5705248)
Supplement: Supplementary file 4 [file IJM-2026-5705248-s004.docx]

**STROBE Statement—Checklist of items for cross-sectional studies**

| **Section/Topic** | **Item** | **Recommendation** | **Reported on page/line number(s)** |
| --- | --- | --- | --- |
| Title and abstract | 1a | Indicate the study design with a commonly used term in the title or the abstract. | Page 1 (title: retrospective laboratory-based study); page 2 (Abstract, Methods). |
| Title and abstract | 1b | Provide in the abstract an informative and balanced summary of what was done and what was found. | Page 2 (Abstract). |
| Introduction | 2 | Explain the scientific background and rationale for the investigation being reported. | Pages 2-3 (Introduction). |
| Introduction | 3 | State specific objectives, including any prespecified hypotheses. | Page 3 (end of Introduction: study objective and primary/exploratory analysis framework). |
| Methods | 4 | Present key elements of study design early in the paper. | Page 3 (Methods - Study design, setting, and data source). |
| Methods | 5 | Describe the setting, locations, and relevant dates, including periods of recruitment, exposure, follow-up, and data collection. | Pages 3-4 (setting and study period, January 1, 2022-December 31, 2025); page 14, Figure 1 legend (data extracted in February 2026). |
| Methods | 6a | Give the eligibility criteria, and the sources and methods of selection of participants. | Page 4 (Specimens and eligibility criteria; anatomical specimen groups and selection of culture-positive records). |
| Methods | 6b | For matched studies, give matching criteria and number of exposed and unexposed. | Not applicable (no matched design). |
| Methods | 7 | Clearly define all outcomes, exposures, predictors, potential confounders, and effect modifiers. Give diagnostic criteria, if applicable. | Pages 4-6 (specimen groups, primary/exploratory analysis groups, isolate categories, AST outcome definitions, and susceptibility denominators). |
| Methods | 8 | For each variable of interest, give sources of data and details of methods of assessment. Describe comparability of assessment methods if there is more than one group. | Pages 3-5 (laboratory information system as data source; microbiological procedures, organism identification, and AST assessment methods). |
| Methods | 9 | Describe any efforts to address potential sources of bias. | Pages 4-6 (unique-patient review, separate exploratory handling of airway isolates, agent-specific AST denominators, and missing-AST handling); page 10 (limitations and potential selection bias). |
| Methods | 10 | Explain how the study size was arrived at. | Page 3 (all 356 eligible laboratory culture records during the defined study period were identified; 136 culture-positive records formed the analytic cohort). |
| Methods | 11 | Explain how quantitative variables were handled in the analyses. If applicable, describe which groupings were chosen and why. | Page 5 (age analyzed as a continuous variable and summarized as mean +/- SD; susceptibility proportions calculated using agent-specific tested denominators; no continuous-variable categorization). |
| Methods | 12a | Describe all statistical methods, including those used to control for confounding. | Pages 5-6 (SPSS version 26; frequencies and percentages, mean +/- SD, exact two-sided Clopper-Pearson 95% CIs, and two-sided Fisher's exact tests). |
| Methods | 12b | Describe any methods used to examine subgroups and interactions. | Pages 5-6 (source-stratified susceptibility analysis comparing primary pleural/deep surgical specimens with exploratory tracheal/airway specimens). |
| Methods | 12c | Explain how missing data were addressed. | Page 6; Supplementary Table S3 (missing AST results defined as not tested; no imputation; testing completeness examined by year and analysis group; agent-specific denominators used). |
| Methods | 12d | If applicable, describe analytical methods taking account of sampling strategy. | Not applicable (all eligible records in the laboratory dataset were assessed; no complex sampling design or sampling weights). |
| Methods | 12e | Describe any sensitivity analyses. | Not performed (no sensitivity analyses). |
| Results | 13a | Report numbers of individuals at each stage of study: potentially eligible, examined for eligibility, confirmed eligible, included, completing follow-up, and analyzed. | Page 6 (Results) and Figure 1 (356 records assessed; 220 culture-negative records excluded; 136 culture-positive records analyzed; 110 primary pleural/deep and 26 exploratory airway specimens). |
| Results | 13b | Give reasons for non-participation at each stage. | Page 6 and Figure 1 (220 records excluded because cultures were negative; no additional duplicate-positive exclusions were required). |
| Results | 13c | Consider use of a flow diagram. | Figure 1 (study flow diagram). |
| Results | 14a | Give characteristics of study participants and information on exposures and potential confounders. | Pages 6-7 (Results); Tables 1-2 (age, sex, culture year, specimen category, isolate type, and microorganism distribution). |
| Results | 14b | Indicate the number of participants with missing data for each variable of interest. | Pages 5-8; Tables 1-5 and Supplementary Tables S1-S3 (core-variable denominators shown; AST missingness reflected by agent-specific N tested and year-specific testing-completeness data). |
| Results | 15 | Report numbers of outcome events or summary measures. | Pages 6-8; Tables 1-5 and Supplementary Tables S1-S3 (culture positivity, organism distribution, and organism-specific susceptibility summaries). |
| Results | 16a | Give unadjusted estimates and, if applicable, confounder-adjusted estimates and their precision. | Pages 6-9; Tables 3-5 and Supplementary Table S1 (susceptibility estimates with exact 95% CIs); Supplementary Table S2 (unadjusted Fisher's exact p values). No adjusted estimates were produced. |
| Results | 16b | Report category boundaries when continuous variables were categorized. | Not applicable (no continuous variables were categorized for analysis). |
| Results | 16c | If relevant, consider translating estimates of relative risk into absolute risk for a meaningful time period. | Not applicable (no relative-risk estimates were reported). |
| Results | 17 | Report other analyses done, e.g., analyses of subgroups and interactions, and sensitivity analyses. | Pages 7-8; Supplementary Tables S2-S3 (exploratory source-stratified susceptibility comparisons and AST-completeness analyses). No sensitivity analyses were performed. |
| Discussion | 18 | Summarize key results with reference to study objectives. | Pages 8-10 (Discussion; results interpreted in relation to the study objectives and external literature). |
| Discussion | 19 | Discuss limitations of the study, taking into account sources of potential bias or imprecision; discuss both direction and magnitude of any potential bias. | Page 10 (Limitations). |
| Discussion | 20 | Give a cautious overall interpretation of results considering objectives, limitations, multiplicity of analyses, results from similar studies, and other relevant evidence. | Pages 8-11 (Discussion, Interpretation and implications, Limitations, and Conclusion; cautious interpretation considering small denominators, exploratory comparisons, and data limitations). |
| Discussion | 21 | Discuss the generalisability (external validity) of the study results. | Page 10 (Interpretation and implications and Limitations: referral-laboratory setting and lack of a defined hospital catchment population limit generalizability). |
| Other information | 22 | Give the source of funding and the role of the funders for the present study and, if applicable, for the original study on which the present article is based. | Page 11 (Funding: no funding was received for this research). |
